# Retention of phytosiderophores by the soil solid phase – adsorption and desorption

**DOI:** 10.1007/s11104-016-2800-x

**Published:** 2016-02-18

**Authors:** M. Walter, E. Oburger, Y. Schindlegger, S. Hann, M. Puschenreiter, S. M. Kraemer, W. D. C. Schenkeveld

**Affiliations:** Department of Environmental Geosciences and Research Network Environmental Science, University of Vienna, Althanstraße 14 (UZA II), 1090 Vienna, Austria; Department of Forest and Soil Sciences, University of Natural Resources and Life Sciences, Konrad Lorenz Strasse 24, 3430 Tulln, Austria; Department of Chemistry, University of Natural Resources and Life Sciences, Muthgasse 18, 1190 Vienna, Austria

**Keywords:** Fe acquisition, Phytosiderophore, Deoxymugineic acid, Rhizosphere, Adsorption, Desorption

## Abstract

**Background and aims:**

Graminaceous plants exude phytosiderophores (PS) for acquiring Fe. Adsorption of PS and its metal complexes to the soil solid phase reduces the FePS solution concentration and hence Fe uptake. In this study we aimed to quantify adsorption, and to determine to what extent adsorption depends on the complexed metal and on soil properties. Furthermore, we examined if adsorption is a reversible process.

**Methods:**

Adsorption and desorption of PS and metal-PS complexes were examined in batch experiments in which the PS 2′-deoxymugineic acid (DMA) and its metal-complexes (FeDMA, CuDMA, NiDMA and ZnDMA) interacted with several calcareous soils.

**Results:**

Adsorption of DMA ligand (0–1000 μM) and metal-DMA complexes (0–100 μM) was linear in the concentration range examined. Adsorption varied by a factor ≈2 depending on the complexed metal and by up to a factor 3.5 depending on the soil. Under field-like conditions (50 % water holding capacity), 50–84 % of the DMA was predicted to be retained to the soil solid phase. Alike adsorption, desorption of metal-DMA complexes is fast (approximate equilibrium within 1 hour). However, only a small fraction of the adsorbed FeDMA (28–35 %) could be desorbed.

**Conclusions:**

Despite this small fraction, the desorbed FeDMA still exceeded the amount in solution, indicating that desorption of FeDMA from soil reactive compounds can be an important process buffering the solution concentration.

**Electronic supplementary material:**

The online version of this article (doi:10.1007/s11104-016-2800-x) contains supplementary material, which is available to authorized users.

## Introduction

Graminaceous plants such as wheat, barley, corn and aerobic rice exude chelating ligands called phytosiderophores (PS) for acquiring iron (Fe) from the soil (Strategy II Fe acquisition) (Marschner et al. 1986; Takagi et al. 1984; Takagi 1976). Upon release into the rhizosphere, PS can bind and solubilize Fe from the soil by forming soluble multidentate Fe complexes that can be readily taken up by graminaceous plants.

For much cultivated graminaceous species including wheat and barley the exudation of PS mainly takes place in the apical root zone as a diurnal pulse release that starts a few hours after the onset of daylight and lasts for 4–6 h (Marschner et al. 1987; Oburger et al. 2014; Takagi et al. 1984). Once exuded, PS participate in geochemical processes that generate a time and Fe-concentration window during which plants can acquire Fe from rhizosphere soil solutions (Schenkeveld et al. 2014b). The processes and factors that influence this window of Fe-uptake include the PS exudation rate, the Fe release rate from the soil, the solubility of the Fe-phases in the soil, the degradation of the PS-ligand, competitive complexation of metals other than Fe, and adsorption of PS-ligand and metal-PS complexes (Mimmo et al. 2014; Reichman and Parker 2005; Schenkeveld et al. 2014b).

Controlled model experiments were instrumental for the elucidation of mechanisms and rates of Fe-mobilization from minerals by PS. Fe mobilization by the PS 2′-deoxymugineic acid (DMA) from the Fe(hydr)oxide mineral goethite was shown to be first order in DMA surface loading (Reichard et al. 2005). The presence of organic ligands like oxalate in such model systems increased the dissolution rate. Furthermore, a fast non-steady state dissolution was found upon addition of DMA when oxalate had been added in advance (Reichard et al. 2005). The oxalate adsorbed and formed a kinetically labile Fe surface species. This Fe could be readily mobilized by DMA (Reichard et al. 2007). Fe mobilization from Fe(hydr)oxide minerals by PS increased with decreasing stability of the Fe(hydr)oxide mineral (Inoue et al. 1993) and decreased when anions like carbonate and phosphate were adsorbed onto the Fe(hydr)oxide mineral surface (Watanabe and Matsumoto 1994). Cesco et al. (2002, 2000) have demonstrated that Fe associated with water extractable humic substances (WEHS) can also serve as an Fe source for PS. Fe mobilization by PS from soil has been demonstrated in several studies (Awad et al. 1988; Schenkeveld et al. 2014a; Takagi et al. 1988; Zhang et al. 1991). In the presence of an excess of PS ligands, the mobilization rate decreased with increasing amount of mobilized Fe (Schenkeveld et al. 2014b).

Contrary to synthetic Fe chelates like iron ethylene diamine-N,N′-bis(hydroxy phenyl acetic acid) (FeEDDHA) and iron ethylene diamine tetra acetic acid (FeEDTA) that are used for micronutrient fertilization, the concentration Fe-PS complexes in soil solution is considerably affected by microbial degradation (Bucheli-Witschel and Egli 2001; Reichman and Parker 2005; Schenkeveld et al. 2012a; Shi et al. 1988; Takagi et al. 1988; Von Wirén et al. 1995). Von Wirén et al. found that Fe acquisition by barley and sorghum grown in nutrient solution was lowered in the presence of microorganisms (Von Wirén et al. 1995) and concluded that efficient Fe acquisition by Strategy II plants from solid substrates is related to the fact that PS release occurs in the apical root zone which has a low population density of rhizosphere microorganisms (Von Wirén et al. 1993). In batch experiments with soil, a lag phase between the addition of PS and the onset of degradation was observed (Schenkeveld et al. 2014b), and depletion of FePS and ZnPS complexes was faster than for CuPS and NiPS complexes.

Apart from Fe, PS have been shown to mobilize other metals including Cu, Ni, Zn, Co, Mn and Cd (Schenkeveld et al. 2014b; Shenker et al. 2001; Takagi et al. 1988; Treeby et al. 1989; Zhang et al. 1991). In a soil environment, these metals compete with Fe for complexation by the PS ligand, reducing Fe mobilization. In a recent study by Schenkeveld et al. (2014b) it was shown that particularly Cu may restrict the time and concentration window of Fe acquisition. In other soils, NiDMA and ZnDMA became the principal DMA-species over time (Schenkeveld et al. 2014a; Schindlegger et al. 2015).

The adsorption of PS has only been explored to a very limited extent so far (Reichman and Parker 2005) and the impact of the ad- and desorption of PS and metal-PS complexes on Fe acquisition is still poorly understood. Although adsorption does not remove the PS ligand from the soil system, it lowers the concentrations of free PS ligand and metal-PS complexes in solution, including the Fe-PS complex which is essential for Fe uptake. Most work on PS adsorption has been done in model systems with Fe(hydr)oxide minerals. Inoue et al. (1993) observed that PS adsorption to Fe(hydr)oxide mineral surfaces decreased with increasing pH, and that from pH 10 onward, adsorption was negligible. Per unit mass, adsorption was higher for minerals with a higher specific surface area. Also Fe-PS complexes adsorbed and the adsorbed fraction of PS decreased with increasing initial PS concentration and with decreasing suspension density. Reichard et al. (2005) also found that adsorption of DMA to goethite decreased with increasing pH with adsorption reaching a maximum around 500 nmol m^−2^ at pH 6 and at around 200 nmol m^−2^ at pH 8. Hiradate and Inoue (1998) demonstrated that adsorption of PS decreased when sulfate or phosphate were adsorbed onto the Fe(hydr)oxide surface. A structured examination of PS adsorption to soil is still lacking and very few studies report on the issue. Hiradate and Inoue (2000) found high PS adsorption in acidic soils. This finding does however not correspond with results from Schenkeveld et al. (2014a), who did not observe substantially more or less PS adsorption in an acidic soil compared to calcareous soils.

Advances in rhizosphere sampling and analytical techniques render it possible to collect pore water samples and to determine the PS concentration therein. In a recent pot trial study with wheat grown on several calcareous soils, Oburger et al. (2014) for the first time reported PS pore water concentrations, which were in the order of 1 μM. It was however unclear how much PS was retained to the solid phase, and to what extent the PS pore water concentration was buffered through desorption. To better understand the effect of PS retention to soil on Strategy II Fe acquisition, a more quantitative understanding of the adsorption and desorption processes is required. Our present study aims to contribute to this understanding.

Firstly, we hypothesize that for conditions under which Strategy II plants grow on soil, more PS is retained to the soil-solid phase than that is present in the pore water. Secondly we hypothesize that the extent to which PS are retained depends on soil characteristics and on the speciation of the PS (i.e. whether it is present as free ligand, or as a certain metal-PS complex). And thirdly, we hypothesize that metal-PS retained by the soil solid phase can become available again through desorption and can buffer the pore water concentration. To test these hypotheses, adsorption and desorption behaviour of metal-DMA complexes was examined in a series of batch and incubation experiments involving seven soils and a range of concentrations of the DMA complexes of Fe, Cu, Zn and Ni.

## Materials and methods

### Materials

#### Soils

Soils were collected from sites in Austria (Lassee and Arnoldstein A), Italy (Bologna), Saudi Arabia (Nadec) and Spain (Santomera and Xeraco). At all sites the top layer was sampled (0–20 cm), and at the Xeraco site, also the layer directly underneath (20–40 cm) was sampled. All are calcareous soils and have been used in previous Fe acquisition studies, both with Strategy I (Schenkeveld et al. 2008) and Strategy II (Oburger et al. 2014) plants. Soils were air-dried and sieved over 2 mm before usage. Selected soil properties are presented in Table 1.

**Table 1:** Selected soil parameters of the soils used in the metal-DMA complex adsorption experiments; in part these data were previously reported in Schenkeveld et al. 2014a

	Water holding capacity (mL kg^−1^)	pH (CaCl_2_)	CaCO_3_ content (g kg^−1^)	Clay content (g kg^−1^)	SOC content (g kg^−1^)	DCB-extraction		AmOx-extraction		DTPA-extractable			
						Al	Fe	Al	Fe	Cu	Fe		
						(g kg^−1^)	(g kg^−1^)	(g kg^−1^)	(g kg^−1^)	(mg kg^−1^)	(mg kg^−1^)	Ni (mg kg^−1^)	Zn (mg kg^−1^)
Xeraco L	620	7.7	147	440	14.2	2.7	17.5	2.1	2.2	3.1	7.5	0.4	5.7
Santomera	500	7.8	500	306	7.3	0.9	10.2	0.6	0.5	1.6	4.9	0.3	0.5
Bologna	610	7.6	150	270	9.1	1.1	9.3	0.6	2.1	3.3	15.5	1.0	0.5
Lassee	540	7.7	138	270	15.5	1.1	5.9	1.7	1.1	1.2	4.8	0.6	0.9
Arnoldstein A	450	7.2	323	235	32.8	2.0	15.6	4.1	3.7	8.3	18	0.7	122
Xeraco T	500	7.5	420	170	28.5	0.9	5.2	1.1	2.5	1.4	75.7	0.5	6.6
Nadec	330	7.6	150	100	8.3	0.5	3.3	0.3	0.3	0.4	9.6	0.2	0.6

#### Ammonium DMA

The ammonium DMA salt used for experimental work was synthesized in accordance with Namba et al. (2007). The DMA salt was over 95 % pure based on NMR analysis, and readily dissolved in water. ^13^C-labelled DMA was synthesized in accordance with Walter et al. (2014) and was used for analytical purposes.

#### Metal-DMA complex solutions

CuDMA and NiDMA solutions were prepared by adding dissolved chloride salts of Cu(II) and Ni(II) to a DMA solution, in a 2 % excess based on a 1:1 stoichiometric ratio to ensure complete complexation of the DMA ligand. FeDMA and ZnDMA solutions were prepared similarly from dissolved Fe(III) and Zn(II) chloride salts, yet with a 60 % excess of DMA ligand. This excess was added because preliminary tests had shown that when Fe and DMA were added in a 1:1 ratio, not all Fe was complexed, and that complete complexation could be accomplished by addition of excess ligand. Furthermore, Fe and Zn were shown to be displaced from DMA complexes by Cu, Ni and Co upon interaction with soil, when the free DMA ligand concentration was low (Schenkeveld et al. 2014b). We chose 60 % excess DMA ligand to prevent this competitive displacement, so that loss from solution can be interpreted as adsorption, which would otherwise not be possible. Metal mobilization from soil by the excess free ligand was accounted for in deriving the adsorption isotherms as described in the Supporting Information. The effect of the excess free ligand on the adsorption behaviour of metal-DMA complexes was tested with NiDMA, but no effect was established.

For preparation of solutions, analytical grade chemicals and ultra-pure water were used.

### Adsorption experiments

Adsorption kinetics of metal-DMA complexes were examined to establish the time required to reach adsorption equilibrium. For this purpose a 30 μM CuDMA solution was added to Santomera soil and samples were taken after 0.5, 1, 2, 4 and 8 h. 2 g L^−1^ NaN_3_ was added as sterilant to prevent biodegradation of the DMA ligand from affecting the CuDMA concentration.

To examine the affinity of metal-DMA complexes for the soil solid phase, soils interacted with FeDMA, CuDMA, NiDMA and ZnDMA solutions. No isotherms were determined for CoDMA, because the redox state of Co complexed by DMA could not be established. Potential undefined mixes of Co(II)DMA and Co(III)DMA would lead to an incorrect determination of the adsorption isotherms.

Santomera soil interacted with 0, 0.25, 0.5, 1, 2.5, 5, 10, 25, 50 and 100 μM metal-DMA solutions, the other soils interacted with 0, 5, 25 and 100 μM metal-DMA solutions. A 150 μM excess of free DMA ligand was applied in adsorption experiments with FeDMA and ZnDMA to avoid the loss of these metal-DMA complexes due to competitive metal displacement by Cu, Ni and Co upon interaction with soil (Schenkeveld et al. 2014b), as discussed above. An interaction time of 2 h for the adsorption equilibrium experiments facilitated accurately reproducible metal mobilization by the added free ligand. No sterilant was added, as test experiments had shown that for the duration of 2 h, biodegradation did not affect the concentration of metal-DMA complexes under our experimental conditions.

All adsorption experiments were carried out in 50 ml polypropylene centrifuge tubes in a soil to solution ratio (SSR) of 1. 10 mM CalCl_2_ was used as background electrolyte. Soils were pre-equilibrated for 2 days at 90 % of the final solution volume containing all electrolyte salt. Samples were placed in an end-over-end shaker rotating at 18 rpm in the dark. After 2 h of interaction, the samples were centrifuged for 3 min at 4500 rpm and filtered over a 0.45 μM cellulose acetate filter (Whatman Aqua 30/0.45 CA). The pH of the filtrates was measured and the filtrates were further analysed (see below). Adsorption experiments were carried out in duplicates.

### Desorption experiment

To examine the desorption kinetics of metal-DMA complexes from the soil solid phase, Santomera soil interacted with 0, 30 and 180 μM DMA solutions containing 10 mM CaCl_2_ and 2 g l^−1^ NaN_3_ in a SSR of 6 (corresponding with 33 % of the water holding capacity (Oburger et al. 2014)) for respectively 1 and 8 h in 200 ml polypropylene pots. The 0 DMA treatment was included as a blank treatment in order to measure DMA-independent metal desorption. Both treatments containing DMA were corrected for DMA-independent metal desorption by subtraction of the metal concentrations in the 0 DMA treatment. After interaction between the soil and the DMA solutions, the soil was transferred to a 500 ml low density polyethylene pot.10 mM CaCl_2_ solution containing 2 g l^−1^ NaN_3_ was added to a final SSR of 0.1 to desorb the metal-DMA complexes from the soil solid phase. The suspension was split into subsamples that were placed in an end-over-end shaker and sampled after respectively 0.25, 0.5, 1, 4 and 24 h. Samples were centrifuged for 3 min at 4500 rpm and filtered over 0.45 cellulose acetate filters. The filtrate was further analysed.

Also the DMA speciation in the pore water after the initial interaction of the DMA solution with Santomera soil (at SSR of 6) was examined. For this purpose, samples were prepared identically to the ones described above. However, after interaction these samples were centrifuged at 7000 rpm in two compartment centrifuge tubes, as described in Schenkeveld et al. (2008). The filtrate was further analysed (see below). In the desorption experiment, release of CoDMA from the soil solid phase was examined, disregarding the redox state of the Co in the CoDMA complex. The desorption experiment was carried out in duplicates.

### Analysis

Metal concentrations (Fe, Zn, Cu, Ni, Co and Mn) in the samples were measured by ICP-MS (Perkin Elmer, ELAN 6100) and ICP-OES (Optima 5300 DV, Perkin Elmer). Samples were acidified with nitric acid prior to analysis. The concentrations of metal-DMA complexes were calculated as the difference between the concentration in the treatment with DMA addition and the corresponding treatment without DMA addition. Schindlegger et al. (2015) observed total dissolved metal concentrations in excess of metal-DMA complex concentrations in the highly metal contaminated Arnoldstein soil. In this study we are assuming that the subtraction of the background metal concentrations (in the absence of DMA) corrects for this, but we did not further measure metal-DMA complex concentrations. For determining an adsorption isotherm for the free DMA ligand, the total DMA ligand concentration was measured by LC-ESI-MS/MS in selected samples, in accordance with Schindlegger et al. (2014).

## Results

### Adsorption kinetics

**Fig. 1:**
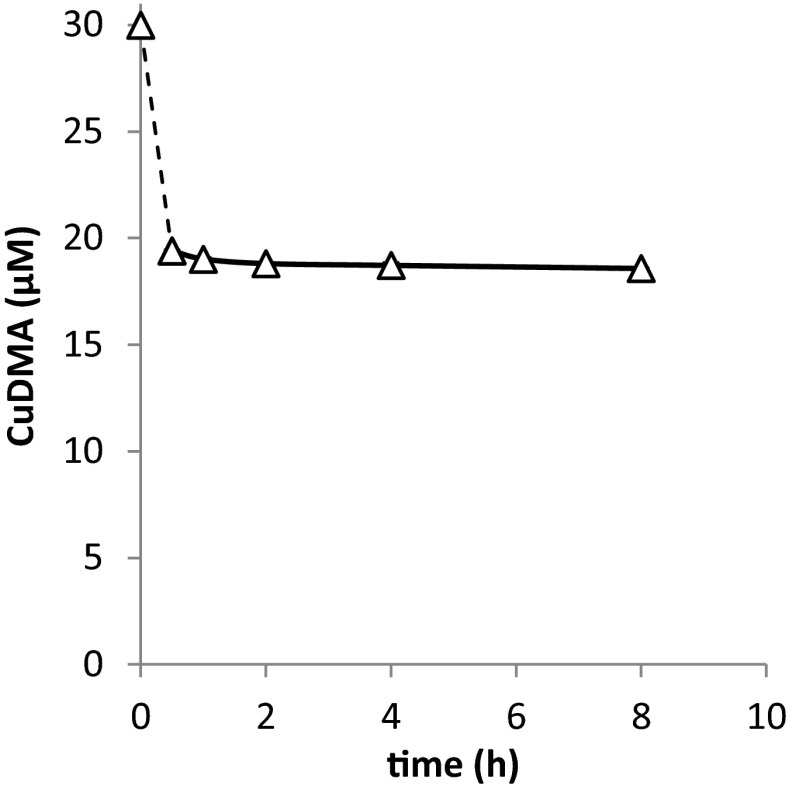
Adsorption kinetics for 30 μM CuDMA interaction with Santomera soil (SSR = 1; 10 mM CaCl_2_; 2 g l^−1^ NaN_3_). Error bars indicate standard deviations

In order to examine the extent to which DMA complexes adsorb to soil, first the time required to reach adsorption equilibrium was examined. This was done for CuDMA adsorption to soil, because on short time scale the CuDMA concentration is relatively unaffected by competition from other metals. The results in Fig. 1 demonstrate that CuDMA adsorption was near instantaneous. It was checked that for NiDMA equilibrium was also reached within 2 h (data not shown). Near instantaneous adsorption of DMA was already suggested in a previous study (Schenkeveld et al. 2014b). Adsorption equilibrium on a comparable timescale was assumed for ZnDMA and FeDMA.

### Adsorption isotherms for metal-DMA complexes to Santomera soil

The data collected from the equilibrium adsorption experiments were used for determining adsorption isotherms. In the experiments with FeDMA and ZnDMA, the 150 μM excess DMA ligand added mobilized 10.7 ± 0.2 μM Fe, 4.4 ± 0.1 μM Cu, 2.0 ± 0.0 μM Zn and 1.2 ± 0.0 μM Ni. This ‘base line metal mobilization’ was unaffected by addition of metal-DMA complexes and could hence be accounted for in determining the adsorption isotherms for FeDMA and ZnDMA. The procedure that was followed for this is described in the Supporting Information and illustrated in SI-Fig. 1.

**Fig. 2:**
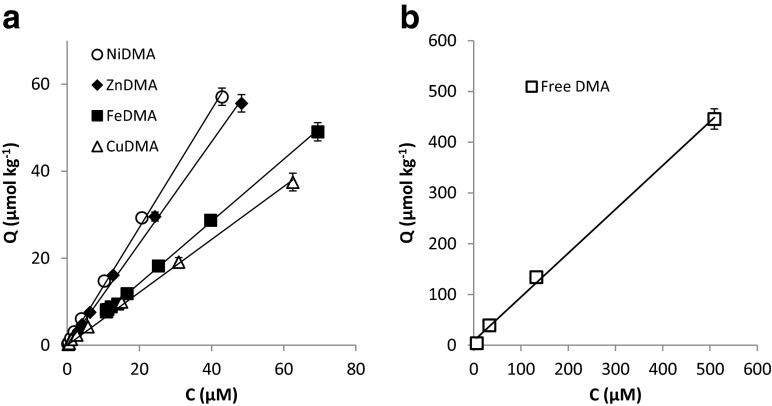
Adsorption isotherms for (**a**) NiDMA, ZnDMA, FeDMA and CuDMA adsorption, and (**b**) free DMA ligand adsorption to Santomera soil. Error bars indicate standard deviations

The adsorption data for FeDMA, CuDMA, NiDMA and ZnDMA to Santomera soil (Fig. 2a) were fit and the isotherms proved linear over the examined concentration range (0–100 μM; Table 2). This implies there were no effects from surface saturation of reactive soil compounds on metal-DMA complex adsorption. Adsorption was highest for NiDMA (slope = 1.35) and lowest for CuDMA (slope = 0.61); so the degree to which the different metal DMA complexes adsorbed varied by approximately a factor 2. Adsorption of ZnDMA (slope = 1.17) was comparable to adsorption of NiDMA, and adsorption of FeDMA (slope = 0.71) was comparable to adsorption of CuDMA.

**Table 2:** Linear fits of the adsorption isotherms of DMA species adsorbing to Santomera soil

Species	Linear fit isotherm	R^2^
FeDMA	0.71*C	0.999
CuDMA	0.61*C	0.998
NiDMA	1.35*C	0.998
ZnDMA	1.17*C	0.998
DMA	0.88*C	0.997

In order to construct an adsorption isotherm for free DMA, we re-interpreted data from a previous study on metal mobilization from Santomera soil by DMA (concentration range: 0.1–1000 μM) (Schenkeveld et al. 2014b). The free DMA ligand concentration was calculated by subtracting the sum of mobilized metals from the total ligand concentration; this approach is feasible in accordance with Schindlegger et al. (2015). By means of the derived adsorption isotherms for metal-DMA complexes, adsorbed amounts of metal-DMA complexes were calculated. Adsorption of CoDMA could be neglected, because of the very low CoDMA solution concentrations. Adsorption of MnDMA was assumed to be comparable to adsorption of ZnDMA – Mn mobilization only occurred at DMA concentrations of 100 μM and higher and did not exceed 2 permille of the amount of DMA added. The adsorbed amount of free DMA ligand was calculated by mass balance. From the adsorbed and solution concentrations of the free DMA ligand, an adsorption isotherm to Santomera soil was constructed (Fig. 2b). Also adsorption of the free ligand proved to increase linearly with the solution concentration; the affinity of the free ligand for the soil solid phase (slope = 0.86) was comparable to that of the metal-DMA complexes (Table 2).

### Adsorption to other soils

For six additional soils that differed distinctively in soil properties (Table 1), adsorption isotherms for the metal–DMA complexes were determined, based on a more limited set of initial concentrations (0, 5, 25 and 100 μM of the metal-DMA complex). Adsorption isotherms for FeDMA and ZnDMA were determined only for Nadec and Lassee soils. For the other soils addition of 150 μM free DMA ligand proved insufficient to ascertain that no competitive metal displacement had occurred.

Similarly to Santomera soil, isotherm data could be fitted linearly (SI-Fig. 2). Slopes of the isotherms differed among the soils, ranging from 0.23 to 0.65 for CuDMA and from 0.34 to 1.18 for NiDMA (SI-Table 1; SI-Fig. 2a&b). These ranges indicate that the affinity of the soil solid phase for CuDMA and NiDMA varied by up to a factor 2.8 and 3.5, respectively. For Nadec and Lassee soil, the slopes of the FeDMA isotherms (respectively 0.43 and 0.45) and ZnDMA isotherms (respectively 0.44 and 0.50) were lower than for Santomera soil (SI-Fig. 1c&d). In contrast to Santomera soil, FeDMA adsorption to Nadec and Lassee soil was comparable to NiDMA and ZnDMA adsorption and not to CuDMA adsorption.

**Fig. 3:**
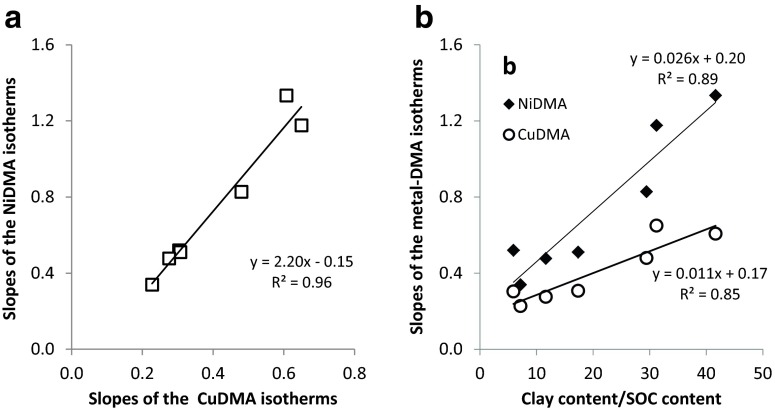
(**a**) The relation between CuDMA and NiDMA adsorption for a range of soils, and (**b**) the relation between CuDMA and NiDMA adsorption and the quotient of the clay content and the SOC content

Differences in soil properties accounted for approximately a factor three difference in adsorption of both CuDMA and NiDMA complexes. The relative degree to which soil properties affected adsorption was very similar for both metal DMA complexes, as illustrated by the linear correlation (R^2^ = 0.96) between the slopes of the NiDMA and CuDMA isotherms of the soils (Fig. 3a). This suggests that the same soil properties are governing the adsorption of both metal-DMA complexes. The slope of the curve indicates that on average adsorption of NiDMA to soil is approximately a factor 2 higher than CuDMA adsorption.

Adsorption was highest in the soils with the highest clay contents (Xeraco L (440 g kg^−1^) and Santomera (310 g kg^−1^)). Correlation of the slopes of the isotherms with clay content of the soils was reasonable (R^2^ = 0.63 for CuDMA and R^2^ = 0.49 for NiDMA; data not shown). A higher correlation was obtained when correlating the slopes of the isotherms with the clay content divided by the organic matter content of the soils (Fig. 3b; R^2^ = 0.84 for CuDMA and R^2^ = 0.89 for NiDMA).

### Desorption of metal-DMA complexes from Santomera soil

Desorption experiments were conducted after reaction of DMA with Santomera soil for 1 h and 8 h. Before the desorption step, the mobilization of metals by DMA during reaction times was observed. The trends in metal mobilization were in agreement with observations from previous studies (Schenkeveld et al. 2014a; Schenkeveld et al. 2014b). CuDMA, NiDMA and CoDMA concentrations increased over time at the expense of FeDMA and ZnDMA concentrations (Table 3). At higher DMA concentration, Fe mobilization was larger and sustained over a longer period of time, because competitive Fe displacement from the FeDMA complex sets in later. For Santomera soil, Cu is the metal that most strongly competes with Fe for complexation by DMA. The fraction of the total mobilized metals accounted for by Cu ranged from approximately 35 % in the 180 μM DMA treatment after 1 h to over 90 % in the 30 μM DMA treatment after 8 h. Metals other than Fe and Cu accounted for less than 10 % in all treatments. The total DMA concentration in soil solution prior to desorption was the same after 1 and 8 h of interaction – for the 180 μM DMA treatment it amounted 36 μM, and for 30 μM DMA treatment 6.6 μM. This accounts for 20–22 % of the DMA added with the treatment, impying that 78–80 % of the total DMA had adsorbed to the soil solid phase. The low remaining DMA fraction in solution is related to the high SSR (6) at which the initial part of the experiment was done, which is representative for field conditions. The relatively high abundance of soil reactive compounds at this SSR enhances DMA sorption.

**Table 3:** Results from the desorption experiment, in which 180 and 30 μM DMA solutions had interacted with Santomera soil in a SSR of 6 for respectively 1 and 8 h. The data presented include solution concentrations prior to desorption, the desorbed amounts expressed in μmol kg^−1^, the ratio between the desorbed amount (in μmol kg^−1^) and the solution concentration prior to desorption (in μM), the equivalent desorbed concentration i.e. the desorbed amount expressed as solution concentration accounting for the SSR, and the predicted adsorbed concentration (in μM) based on the solution concentration prior to adsorption and the adsorption isotherms presented in Table 2

	180 μM DMA					30 μM DMA				
	Solution concentration	Desorbed amount	Ratio des. am./sol. conc.	Equivalent desorbed concentration	Predicted equiv. adsorbed conc.	Solution concentration	Desorbed amount	Ratio des. am./sol. conc.	Equivalent desorbed concentration	Predicted equiv. adsorbed conc.
	(μM)	(μmol kg^−1^)	(l kg^−1^)	(μM)	(μM)	(μM)	(μmol kg^−1^)	(l kg^−1^)	(μM)	(μM)
1 h interaction										
FeDMA	20.3 ± 0.4	5.1 ± 0.2	0.25	30.7 ± 1.3	86.5	1.0 ± 0.4	b.d.			4.2
CuDMA	13.4 ± 0.2	6.7 ± 0.1	0.50	39.9 ± 0.8	48.9	5.5 ± 0.6	2.7 ± 0.1	0.49	16.1 ± 0.7	20
NiDMA	0.6 ± 0.2	0.7 ± 0.1	1.17	4.4 ± 0.6	5.1	b.d.	b.d.			
CoDMA	b.d.	b.d				b.d.	b.d			
ZnDMA	1.6 ± 0.5	1.9 ± 0.1	1.2	11.2	10.9	0.13 ± 0.06	b.d.			0.9
Total	35.8 ± 0.7	14.4 ± 0.3		86.3	151.4	6.6 ± 0.7	2.7 ± 0.1		16.1 ± 0.7	25.2
Total solution + predicted equivalent adsorbed concentration:					187.2 μM					31.8
8 h interaction										
FeDMA	9.9 ± 0.6	2 ± 0.2	0.20	12	42.2	0.5 ± 0.1	b.d.			2.1
CuDMA	22.8 ± 0.5	8.8 ± 0.3	0.39	52.8	83.4	6.1 ± 0.5	2.8 ± 0.1	0.46	16.8 ± 0.6	22.3
NiDMA	1.7 ± 0.1	1.5 ± 0.0	0.88	8.9	13.5	b.d.	b.d.			
CoDMA	0.6 ± 0.1	0.5 ± 0.1	0.77	2.7		b.d.	b.d.			
ZnDMA	1.1 ± 0.0	1.3 ± 0.0	1.23	8.1	7.7	b.d.	b.d.			
Total	36 ± 0.8	14.1 ± 0.4		84.4	146.8	6.6 ± 0.5	2.8 ± 0.1		16.8 ± 0.6	24.3
Total solution + predicted equivalent adsorbed concentration:					182.8					30.9

**Fig. 4:**
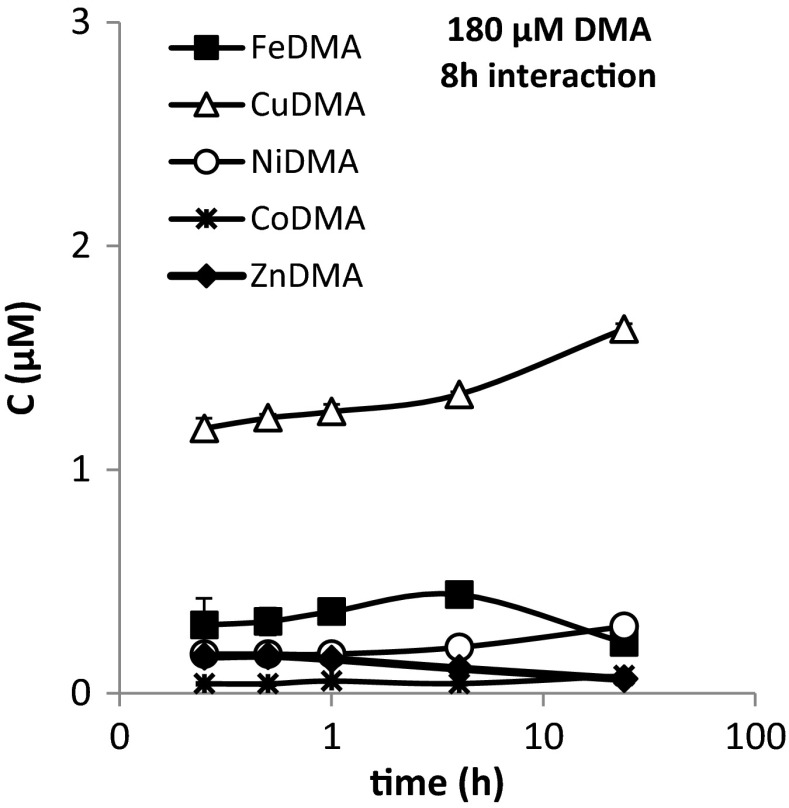
Desorption kinetics of metal-DMA species from Santomera soil in 10 mM CaCl_2_ extract. A 180 μM DMA solution had interacted with Santomera soil for 8 h at SSR = 6. Subsequently the soil was extracted with 10 mM CaCl_2_ at SSR = 0.1. The reported concentrations are those measured in the extract. Error bars indicate standard deviations

After interaction, desorption kinetics were examined using 10 mM CaCl_2_ as extractant. For the 180 μM DMA/ 8 h interaction treatment the metal-DMA concentrations in the extracts are presented as a function of time in Fig. 4 (for other treatments in SI-Fig. 3). The concentrations of the metal-DMA complexes were reasonably similar for the first three time points (up to 1 h). This indicates that desorption of metal-DMA complexes is near instantaneous. For the later time points (4 h and particularly 24 h), the concentrations drift a bit more. However, the increase in CuDMA, NiDMA and CoDMA concentrations and simultaneous decrease in FeDMA and ZnDMA concentration imply that this drift is related to competitive metal exchange rather than to desorption. Because desorption kinetics appear to be very fast and the influence of metal exchange on the metal-DMA complex concentrations increased with extraction time, the desorption data after 1 h were considered most suitable for constructing a mass balance.

The metal-DMA complex concentrations in the extracts were corrected for the solution concentrations before extraction and back-calculated to desorbed concentrations in μmol per kg soil (Table 3). Subsequently, the ratios between desorbed and solution concentration were calculated (Table 3) and compared to the slopes of the adsorption isotherms. Because the amounts of metal-DMA complex still adsorbed after equilibrium was reached in the desorption experiment were disregarded, the desorbed amounts underestimated the initially adsorbed amounts by 7 % (CuDMA) to 13 % (NiDMA), as calculated using the adsorption isotherms presented in Fig. 1 and Table 2. Hence, the ratio desorbed concentration over solution concentration should be an underestimation by a similar percentage, compared to slopes of the isotherms.

Comparison of the ratios (desorbed/solution; Table 3) with the slopes of the isotherms (Table 2) shows that for ZnDMA they are in fair agreement (1.20–1.23 versus 1.17). For NiDMA and CuDMA the agreement is reasonable (0.39–0.50 versus 0.61 for CuDMA and 0.88–1.17 versus 1.35 for NiDMA), but particularly for FeDMA (0.20–0.25 versus 0.71) the deviation is large. Apart from ZnDMA, there is a structural underestimation of the adsorbed fraction (also after correction for the remaining adsorbed fraction after desorption). This suggests that, although desorption is fast, it is not complete; particularly for FeDMA a substantial fraction appears to remain retained to the soil solid phase. This would imply that adsorption of metal-DMA complexes is only in part reversible, and that, at least to a certain extent, adsorption of metal-DMA should be considered a permanent loss term in view of plant Fe acquisition. Nonetheless, in the 180 μM DMA treatments, the desorbed amounts of FeDMA back-calculated to molar concentrations exceeded the solution concentrations by a factor 1.2 to 1.5 (20.3 μM in solution versus 30.7 μM desorbed after 1 h and 9.9 μM in solution versus 12.0 μM desorbed after 8 h; Table 3). This demonstrates that for field-like SSR values, replenishment of the FeDMA concentration through desorption from the soil solid phase during FeDMA uptake by the plant may substantially contribute to Fe acquisition. For all other metal-DMA complexes the ratio between amount desorbed and in solution was higher: 2.3–3 for CuDMA, 5.2–7.3 for NiDMA, 7.0–7.4 for ZnDMA, and 4.5 for CoDMA. The overall ratio for all metal-DMA complexes was 2.3–2.5, which indicates that under environmentally relevant conditions most of the potentially bioavailable DMA is retained to the soil solid phase.

Predictions of adsorbed metal-DMA concentrations (expressed as molar concentrations in solution) prior to desorption were made, based on the metal-DMA pore water concentrations and the slopes of the corresponding adsorption isotherms (Table 2 and 3). As expected, the relative differences between the predicted values and the desorbed amounts were largest for FeDMA. For all treatments, the sum of the solution concentrations plus the sum of predicted adsorbed concentrations corresponded well with the DMA concentration that was applied – deviations were in all cases smaller than 6 % of the added amount. Adsorbed CoDMA was not accounted for because no adsorption isotherms were determined, but its contribution to overall DMA speciation was relatively small. The free DMA concentration in the pore water prior to desorption was not determined, but the consistent mass balance after 1 and 8 h suggests that already after 1 h the free DMA concentration was negligible. The closed mass balances support that adsorption isotherms for DMA complexes can be successfully applied for predicting DMA speciation over the solid and solution phase under field-like conditions. Based on the adsorption isotherms to Santomera soil (Fig. 2, Table 2) and a SSR of 6, the following ratios between amount adsorbed and amount in solution were predicted: 3.6 for CuDMA, 4.3 for FeDMA, 8.5 for NiDMA, 6.8 for ZnDMA.

## Discussion

The linear adsorption of the free DMA ligand and metal-DMA complexes to soil observed in this study do not correspond with the results from studies in which FePS adsorption to Fe(hydr)oxide minerals was studied (Inoue et al. 1993; Reichard et al. 2005). In these studies the slope of the adsorption isotherm decreased with increasing solution concentration, eventually approaching a plateau, implying a sorption maximum. In the present study we did not observe such surface saturation effects. The bending of the isotherms already started within the PS concentration range applied in this study (0–100 μM), and the slopes of the isotherms (with adsorption expressed per mass unit) were considerably lower compared to soil, except for ferrihydrite. This difference in adsorption behaviour between soil and model systems may be caused by the diversity in reactive surfaces in the soil and the interaction between soil reactive compounds. The presence of reactive compounds with a high specific surface area, like clay minerals, may contribute to larger slope of the adsorption isotherm compared to most Fe(hydr)oxide minerals.

Linear adsorption to soil has been previously found for other chelate complexes including racemic and meso o,o-FeEDDHA. The slope of the isotherms was considerably smaller than for the metal-DMA complexes, indicating a lower affinity for the soil solid phase (Schenkeveld et al. 2010).

Upon interaction with soil, reactive soil constituents other than Fe(hydr)oxides can also interact with metal-DMA complexes contributing to overall adsorption. Results from this study suggest that particularly clay minerals may be important in this respect. Furthermore, other soil constituents like soil organic matter can interact with Fe(hydr)oxide and clay mineral phases, altering their surface properties and affinity for metal-DMA complexes. For FeEDDHA it was shown that adsorption of humic acid onto goethite strongly decreased adsorption compared to adsorption to goethite only (Schenkeveld et al. 2012b). Alike FeEDDHA, metal-DMA complexes have a net negative charge, and adsorption of negatively charged humic substances onto Fe(hydr)oxide phases in the soil will contribute to a lower affinity of metal DMA complexes for the Fe(hydr)oxide surfaces due to a decreased electrostatic attraction or an increased electrostatic repulsion. This effect is similar to the observation by Hiradate and Inoue (1998) that PS adsorption decreased when the anions sulphate and phosphate were adsorbed on the Fe(hydr)oxide surface.

The observed higher correlation between metal-DMA adsorption and clay content divided by organic matter content compared to clay content only may indicate that organic matter makes adsorption of anionic metal-PS complexes to clay minerals less favourable. Soil organic matter has been shown to associate with and adsorb to clay minerals in the soil (Pronk et al. 2012). Zhuang and Yu (2002) demonstrated that this association can lower the zeta-potential of clay mineral surfaces and make adsorption of anions less favourable. Possibly, competitive or charge effects resulting from soil organic matter adsorption to clay decrease adsorption of DMA complexes.

Studies examining adsorption of ligands and metal-complexes to soils may help to gain a macroscopic understanding of the overall retention of such compounds in soils. By combining data on adsorption and soil properties, an impression of the relevance of specific reactive soil constituents with respect to adsorption may be obtained. However, for processes like ligand-promoted dissolution of Fe (Reichard et al. 2005), and competitive metal displacement (Schenkeveld et al. 2012b) that are or can be surface controlled, it is not the overall adsorption that is of relevance, but the adsorption to specific reactive surfaces or sites. Therefore, the fact that clay minerals appear to constitute a quantitatively important adsorption surface does not imply that the rate of these processes increases with increasing clay content of a soil. Measuring the loss from solution for adsorption measurement does not provide direct information about the surface speciation. The mechanism by which metal-PS complexes and free PS ligand adsorb to clay and other soil constituents as well as the related surface species are yet unelucidated and should be further examined. Considering that both complex and clay minerals are negatively charged, cation bridging may play a role, as was suggested for adsorption of EDDHA and its metal complexes to montmorillonite clay (Siebner-Freibach et al. 2004).

The different extent to which DMA complexes of different metals adsorb to the same soil may be related to a number of factors, including differences in complex stability (Murakami et al. 1989), differences in charge of the PS complex at soil-pH (von Wirén et al. 2000), differences in complex geometry (Kato et al. 2011), etc. FeDMA and CuDMA have higher 1:1 stability constants than ZnDMA and NiDMA, and adsorbed to a lesser extent to Santomera soil. However, NiDMA has a higher stability constant than ZnDMA, but appears to adsorb to a slightly higher degree. This indicates that, to some degree, stability constants correlate with the extent to which metal-PS complexes adsorb, but that the stability constant is not the sole characteristic of the complex that determines its affinity for soil reactive compounds. Furthermore, von Wirén et al. (2000) demonstrated that at pH 7 (close to the pH values of the soils used) the net negative charge of FePS complexes was larger than for ZnPS complexes, leading to a larger charge repulsion between FePS complexes and negatively charged soil reactive compounds, and, potentially, to less adsorption.

Recently, Oburger et al. (2014) reported on a pot trial experiment with wheat, in which PS concentrations in the pore water of various soils were found to be in the lower micromolar range. These concentrations fall well within the range of the linear adsorption isotherms for free DMA and metal-DMA that were determined in this study, implying that under environmental conditions no surface saturation should be expected.

By means of the range in slopes of the adsorption isotherms for the different soils, an estimation can be made of the variation in adsorbed fraction under natural conditions for the individual metal-DMA complexes. The adsorbed fraction can be calculated by taking the adsorption isotherm (Eq. 1) and multiplying the adsorbed content (Q) with the soil solution ratio (SSR), to obtain the adsorbed concentration (C_S_) (Eq. 2). By mass balance, the total concentration equals the solution concentration plus the adsorbed concentration (Eq. 3), and the adsorbed fraction equals the adsorbed concentration divided by the total concentration (Eq. 4).

1 $$ Q=\upalpha \bullet {C}_L $$

Q = adsorbed content (mol kg^−1^), α = adsorption coefficient (kg l^−1^) – i.e. slope of the adsorption isotherm, C_L_ = solution concentration (mol l^−1^)

2 $$ {C}_S=Q\bullet SSR=\alpha \bullet {C}_L\bullet SSR $$

C_S_ = adsorbed concentration (mol l^−1^), SSR = soil to solution ratio (l kg^−1^)

3 $$ {C}_T={C}_S+{C}_L={C}_L\bullet \left(1+\alpha \bullet SSR\right) $$

C_T_ = total concentration (mol l^−1^)

4 $$ {f}_S=\frac{C_S}{C_T}=\frac{\alpha \bullet SSR}{1+\alpha \bullet SSR} $$

f_S_ = adsorbed fraction

For the soils included in this study, the adsorbed fraction of metal-DMA complexes was calculated for 50 % of the water holding capacity (Table 1), which can be considered representative for field conditions. Because clay soils tend to have a higher water holding capacity than sandy soils, 50 % of the water holding capacity corresponds with a lower SSR for clay soils than for sandy soil. Combined with the fact that clay soils generally had higher adsorption coefficients than sandy soils, setting the SSR to 50 % of the water holding capacity had a levelling effect on the variation in adsorbed fractions among soils. For CuDMA, the adsorbed fraction amounts 50–71 %, and for NiDMA 60–84 %; for the three soils for which the adsorption isotherms for FeDMA and ZnDMA were determined, the adsorbed fraction ranges from respectively 63–74 % and 65–82 %. For these conditions, the amount of CuDMA adsorbed exceeds the amount in solution by a factor 1.0–2.4; for NiDMA the adsorbed amount is a factor 1.5–5.3 larger, for FeDMA a factor 1.7–2.8 and for ZnDMA a factor 1.9–4.6. Oburger et al. (2014) reported DMA pore water concentrations up to 1.44 μM. Depending on the DMA solution speciation, which was not determined in the aforementioned study, this pore water concentration corresponds with a total DMA concentration (solution and solid phase; expressed as solution concentration) of 4.8 (exclusively CuDMA) to 9.1 μM (exclusively NiDMA).

These calculations confirm our hypothesis that, once exuded into soil solution, most PS will be retained to the soil solid phase, and that the extent to which it is retained depends on both the complexed metal and on the characteristics of the soil. The desorption experiment showed that only part of the adsorbed metal-DMA complexes became available again through desorption; particularly for FeDMA complexes, adsorption appears, in part, to be a permanent sink term on the timescales examined. Nevertheless, a considerable amount of FeDMA could be desorbed, exceeding the amount that was in solution. Possibly, adsorption protects the PS ligand from microbial degradation, and desorption of metal-PS complexes from soil reactive compounds may mitigate the decline in PS soil solution concentration as a result of microbial degradation.

Adsorption of the free DMA ligand and metal-DMA complexes to soil is proportional to the solution concentration, and can therefore be described with a linear adsorption isotherm. The extent to which metal-DMA complexes adsorbed varied by up to a factor 2, depending on the chelated metal. NiDMA had the highest tendency to adsorb and CuDMA the lowest. Soil properties influenced adsorption of metal-DMA complexes; adsorption was highest in the soils with the highest clay content, and the slopes of the adsorption isotherms correlated well with the ratio of clay content over organic matter content of the soils. Overall variation in adsorption among the tested soils for the individual metal-DMA complexes was approximately a factor 2.8 to 3.5. Under field-like conditions (50 % water holding capacity), 50–84 % of the DMA was predicted to be retained to the soil solid phase. The kinetics of desorption of metal DMA-complexes from soil were fast, but desorption was not complete. The fraction of metal DMA complexes that would detach from soil reactive compounds would for the large part do so within 0.25 h. However, FeDMA (appr. 28–35 %) and to a lesser degree CuDMA (63–82 %) and NiDMA (66–86 %) would only desorb to a limited extent. For all three metal DMA complexes, the desorbable fraction decreased with time. Despite that the fraction of FeDMA that desorbed was small, still the desorbed amount exceeded the amount in solution. This indicates that desorption of FePS from soil reactive compounds could be an important process buffering the solution concentration and may help to prolong the time-span that PS enhance Fe availability in soil.

## Electronic supplementary material

ESM 1 (DOCX 154 kb)
