# Supplementary material for: Retention of phytosiderophores by the soil solid phase – adsorption and desorption
Source: Plant Soil. 2016 Feb 18;404:85–97. doi: 10.1007/s11104-016-2800-x (PMC4908159; doi:10.1007/s11104-016-2800-x)
Supplement: Supplementary file 1 — (DOCX 154 kb) [file 11104_2016_2800_MOESM1_ESM.docx]

SI-Table 1: Fitted adsorption isotherms for CuDMA, NiDMA, FeDMA and ZnDMA for various soils.

| Species | Soil | Linear fit isotherm | R^2^ |
| --- | --- | --- | --- |
| CuDMA | Xeraco L | 0.65*C | 0.997 |
|  | Bologna | 0.48*C | 0.998 |
|  | Lassee | 0.31*C | 0.995 |
|  | Xeraco T | 0.30*C | 0.999 |
|  | Nadec | 0.28*C | 0.994 |
|  | Arnoldstein A | 0.23*C | 0.995 |
| NiDMA | Xeraco L | 1.18*C | 0.996 |
|  | Bologna | 0.83*C | 0.996 |
|  | Lassee | 0.51*C | 1.000 |
|  | Xeraco T | 0.52*C | 0.998 |
|  | Nadec | 0.48*C | 0.997 |
|  | Arnoldstein A | 0.34*C | 0.999 |
| FeDMA | Lassee | 0.45*C | 0.999 |
|  | Nadec | 0.43*C | 1.000 |
| ZnDMA | Lassee | 0.50*C | 0.996 |
|  | Nadec | 0.44*C | 0.999 |

SI-Figure 1: Calculation of FeDMA and ZnDMA isotherms from the measured solution concentrations.

The excess of 150 µM DMA that was added with treatments containing FeDMA and ZnDMA also chelated Fe and Zn from the soil which was partly mobilized into solution and partly adsorbed. To account for the adsorbed FeDMA and ZnDMA in constructing the adsorption isotherm, the following procedure was applied (described for FeDMA).

The adsorbed concentration related to the FeDMA that was added to the soil was determined by calculating the difference in FeDMA solution concentration between the treatment in which FeDMA + free DMA (x µM FeDMA + 150 µM free DMA) was added and the treatment in which only free DMA was added (0 µM FeDMA + 150 µM free DMA). This difference was subtracted from the FeDMA concentration added to obtain the adsorbed concentration related to the FeDMA that had been added to the soil.

FeDMA_ads added(t=t)_ = FeDMA_added(t=0)_ –(FeDMA_iFeDMA+DMA(t=t)_ – FeDMA_only DMA(t=t)_)

This adsorbed FeDMA related to the FeDMA that had been added with the treatment was plotted against the FeDMA solution concentration. In SI-Figure 1 this series is referred to as FeDMA measured data. The data was fitted linearly. Because an adsorption isotherm should start in the origin, the intercept with the y-axis should equal 0. In fact there is a negative intercept, the absolute value of which corresponds with FeDMA adsorption in the 150 µM DMA ligand treatment. In order to obtain the FeDMA adsorption isotherm, the fitted line should be transposed along the y-axis until it passes through the origin, in order to account for FeDMA adsorption resulting from the addition of free DMA ligand. The same procedure was followed for determining the ZnDMA isotherms.

SI-Figure 2: adsorption isotherms for CuDMA, NiDMA, FeDMA and ZnDMA for various soils. 10 mM CaCl_2_ was used as background electrolyte.

SI-Figure 3: Desorption kinetics of metal-DMA species from Santomera soil in 10 mM CaCl_2_ extract. Santomera soil had interacted at SSR = 6 with a) a 180 µM DMA solution for 1 hour; b) a 30 µM DMA solution for 1 hour, and c) a 30 µM DMA solution for 8 hours. Subsequently the soil was extracted with 10 mM CaCl_2_ at SSR = 0.1. The reported concentrations are those measured in the extract. Error bars indicate standard deviations.
